# NLRP3 inflammasome and its inhibitors: a review

**DOI:** 10.3389/fphar.2015.00262

**Published:** 2015-11-05

**Authors:** Bo-Zong Shao, Zhe-Qi Xu, Bin-Ze Han, Ding-Feng Su, Chong Liu

**Affiliations:** Department of Pharmacology, Second Military Medical UniversityShanghai, China

**Keywords:** NLRP3 inflammasome, inhibitor, autophagy, MCC950, BHB, interferon

## Abstract

Inflammasomes are newly recognized, vital players in innate immunity. The best characterized is the NLRP3 inflammasome, so-called because the NLRP3 protein in the complex belongs to the family of nucleotide-binding and oligomerization domain-like receptors (NLRs) and is also known as “pyrin domain-containing protein 3”. The NLRP3 inflammasome is associated with onset and progression of various diseases, including metabolic disorders, multiple sclerosis, inflammatory bowel disease, cryopyrin-associated periodic fever syndrome, as well as other auto-immune and auto-inflammatory diseases. Several NLRP3 inflammasome inhibitors have been described, some of which show promise in the clinic. The present review will describe the structure and mechanisms of activation of the NLRP3 inflammasome, its association with various auto-immune and auto-inflammatory diseases, and the state of research into NLRP3 inflammasome inhibitors.

## Introduction

The mammalian immune system defends against internal and external threats using innate immunity and adaptive immunity (Neill et al., 2010). The innate immune response relies on pattern-recognition receptors (PRRs) to target pathogenic microbes and other endogenous or exogenous pathogens. PRRs are expressed mainly in immune and inflammatory cells such as monocytes, macrophages, neutrophils, and dendritic cells (DCs) (Schroder and Tschopp, 2010; Fullard and O’Reilly, 2015). They present antigens to the adaptive immune system to generate long-lasting protection (Alexandre et al., 2014). Pathogen-associated molecular patterns (PAMPs), which are antigens common to a given group of pathogens (Medzhitov, 2009; Abderrazak et al., 2015b), are normally recognized by at least three PRRs: Toll-like receptors (TLRs), C-type lectins (CTLs), and Galectins (Bourgeois and Kuchler, 2012; Dzopalic et al., 2012). The innate immune system is evolutionarily conserved across vertebrates and invertebrates, which means that both human and animal studies can provide valuable insights into innate immunity (Dai et al., 2015).

A newly identified PRR, first described in detail in 2002, is the inflammasome (Martinon et al., 2002; Gentile et al., 2015; Jorgensen and Miao, 2015; Sanders et al., 2015). Numerous inflammasomes have been identified, including NLRP1, NLRP2, NLRP3, double-stranded DNA (dsDNA) sensors absent in melanoma 2 (AIM2) and NLRC4 (Ozaki et al., 2015). The best characterized is the NLRP3 inflammasome, so named because the NLRP3 protein in the complex belongs to the family of nucleotide-binding and oligomerization domain-like receptors (NLRs) and is also known as “pyrin domain-containing protein 3” (Inoue and Shinohara, 2013b; Eigenbrod and Dalpke, 2015). In addition to the NLRP3 protein, the NLRP3 inflammasome also contains adapter protein apoptosis-associated speck-like protein (ASC) and procaspase-1 (Inoue and Shinohara, 2013a; Ito et al., 2015). Interactions among these three proteins tightly regulate inflammasome function in order to ensure immune activity only when appropriate.

In the absence of immune activators, an internal interaction occurs between the NACHT domain and leucine-rich repeats (LRRs), suppressing the interaction between NLRP3 and ASC, thus preventing assembly of the inflammasome (Inoue and Shinohara, 2013a). In the presence of immune activators such as PAMPs, danger-associated molecular patterns (DAMPs), other exogenous invaders or environmental stress, NLRP3 opens up and allows interaction between the pyrin domains (PYDs) in NLRP3 and ASC. Subsequently the caspase recruitment domain (CARD) of ASC binds to the CARD domain on procaspase-1, giving rise to the NLRP3 inflammasome. Formation of this complex triggers procaspase-1 self-cleavage, generating the active caspase-1 p10/p20 tetramer and inducing the conversion of proinflammatory cytokines interleukin (IL)-1β and IL-18 from their immature “pro” forms to active forms that are secreted. Formation of the inflammasome also triggers a process of inflammation-related cell death termed pyroptosis (Willingham et al., 2009; Schroder and Tschopp, 2010; Zhong et al., 2013a; Jorgensen and Miao, 2015).

## Activation Of The Nlrp3 Inflammasome

### Models of NLRP3 Inflammasome Activation

**FIGURE 1:**
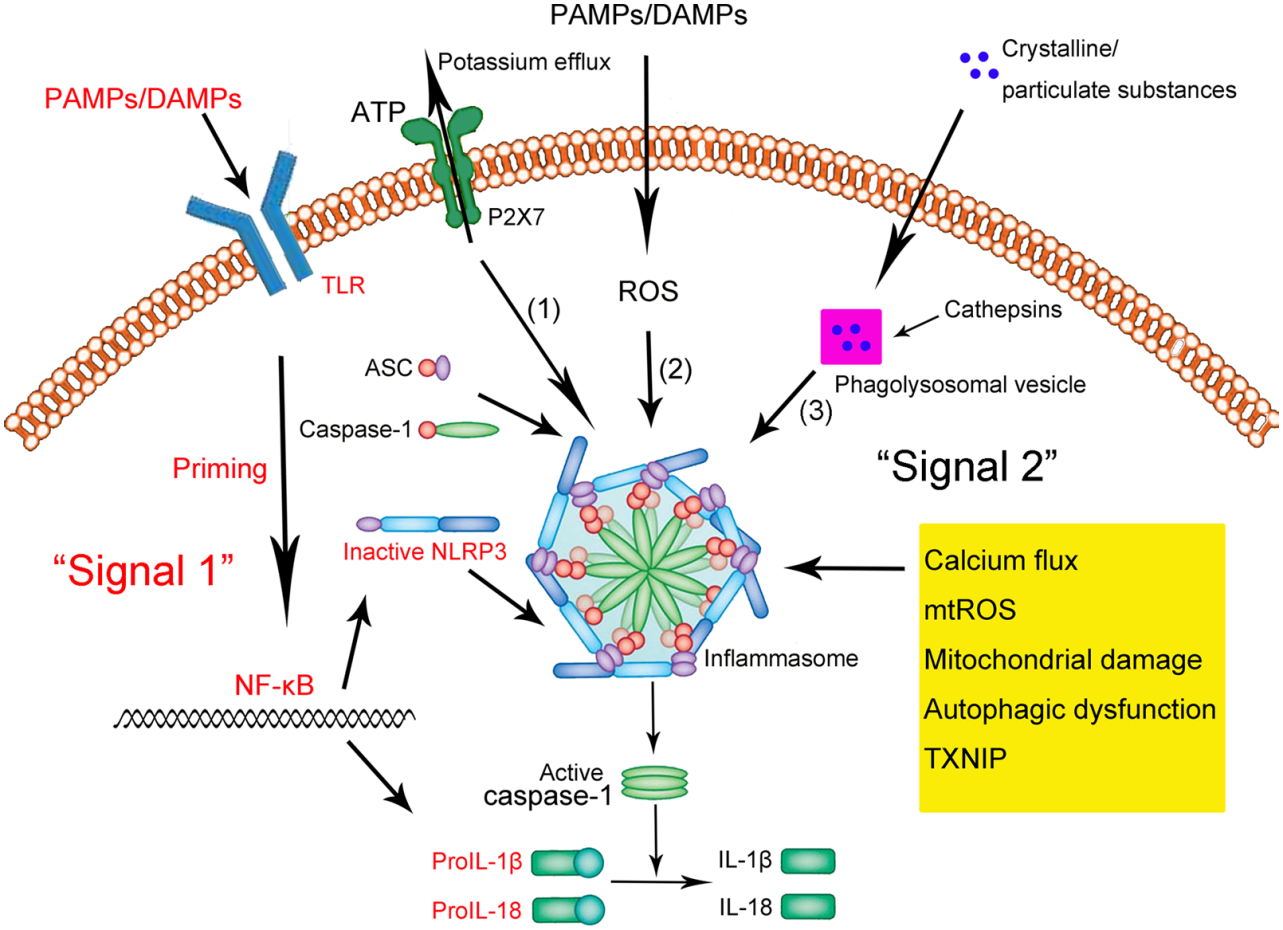
**Schematic illustration of the NLRP3 inflammasome activation.** Upon exposure to pathogen-associated molecular patterns (PAMPs) or danger-associated molecular patterns (DAMPs), Toll-like receptors (TLRs) are phosphorylated and subsequently activate NF-κB. In the nucleus, NF-κB promotes the transcription of NLRP3, proIL-1β, and proIL-18, which, after translation, remain in the cytoplasm in inactive forms. Thus, this signal (depicted in red as “Signal 1”) is a priming event. A subsequent stimulus (shown as “Signal 2” in black) activates the NLRP3 inflammasome by facilitating the oligomerization of inactive NLRP3, apoptosis-associated speck-like protein (ASC), and procaspase-1. This complex, in turn, catalyzes the conversion of procaspase-1 to caspase-1, which contributes to the production and secretion of the mature IL-1β and IL-18. Three models have been proposed to describe the second step of inflammasome activation: (1) Extracellular ATP can induce K^+^/potassium eﬄux through a purogenic P2X7-dependent pore, which, leads to the assembly and activation of the NLRP3 inflammasome. Calcium flux is also involved in this process. (2) PAMPs and DAMPs trigger the generation of ROS that promote the assembly and activation of the NLRP3 inflammasome. (3) Phagocytosed environmental irritants form intracellular crystalline or particulate structures leading to lysosomal rupture (magenta box) and release of lysosomal contents like cathepsin B. These induce NLRP3 inflammasome assembly and activation. In addition, other factors and mechanisms have been implicated in the assembly and activation of the NLRP3 inflammasome, including mitochondrial damage, autophagic dysfunction, and thioredoxin-interacting protein (TXNIP).

The NLRP3 inflammasome is present primarily in immune and inflammatory cells following activation by inflammatory stimuli; these cells include macrophages, monocytes, DCs, and splenic neutrophils (Guarda et al., 2011b; Zhong et al., 2013a). Activation of the NLRP3 inflammasome appears to occur in two steps (Zhong et al., 2013a; Sutterwala et al., 2014; Ozaki et al., 2015; **Figure 1**). The first step involves a priming or initiating signal in which many PAMPs or DAMPs are recognized by TLRs, leading to activation of nuclear factor kappa B (NF-κB)-mediated signaling, which in turn up-regulates transcription of inflammasome-related components, including inactive NLRP3, proIL-1β, and proIL-18 (Bauernfeind et al., 2009; Franchi et al., 2012, 2014). This priming step is often studied *in vitro* using lipopolysaccharide (LPS; Park et al., 2015). The second step of inflammasome activation is the oligomerization of NLRP3 and subsequent assembly of NLRP3, ASC, and procaspase-1 into a complex. This triggers the transformation of procaspase-1 to caspase-1, as well as the production and secretion of mature IL-1β and IL-18 (Kim et al., 2015; Ozaki et al., 2015; Rabeony et al., 2015).

Three models have been proposed to describe the second step of inflammasome activation, as described in detail by Schroder and Tschopp (2010) (shown in **Figure 1**). Briefly, all models assume that NLRP3 does not directly interact with exogenous activators, consistent with its ability to sense various pathogens. In the first model, extracellular adenosine triphosphate (ATP), which acts as an NLRP3 agonist, induces K^+^ eﬄux through a purogenic P2X7-dependent pore consisting of a pannexin-1 hemichannel. This process leads to NLRP3 inflammasome activation and assembly. Consistent with this model, K^+^ eﬄux is a major activator of the NLRP3 inflammasome, while extracellular ATP and pore-forming toxins are the major triggers of IL-1β secretion by the inflammasome (Hari et al., 2014; Liu et al., 2014; Ketelut-Carneiro et al., 2015; Schmid-Burgk et al., 2015). Fluxes of intracellular and endoplasmic reticulum (ER)-related Ca^2+^ may also activate the NLRP3 inflammasome (Hussen et al., 2012; Zhong et al., 2013b; Shenderov et al., 2014).

In the second model, all known PAMPs and DAMPs, including the activators mentioned above, trigger the generation of reactive oxygen species (ROS), which in turn induce assembly of the NLRP3 inflammasome. For example, damage to NADPH oxidase and other oxidative systems by mitochondrial ROS can activate the inflammasome (van Bruggen et al., 2010; Crane et al., 2014; Lawlor and Vince, 2014; Rajanbabu et al., 2015).

In the third model, assembly and activation of the NLRP3 inflammasome is thought to be triggered by environmental irritants (such as silica, asbestos, amyloid-β, and alum) which form crystalline or particulate structures when engulfed by phagocytes. These aggregates cause lysosomal rupture and release of lysosomal contents via a mechanism mediated by cathepsin B. Consistent with this model, crystalline stimuli such as silica are major triggers of IL-1β secretion by the inflammasome.

Other factors can also activate the NLRP3 inflammasome. These include mitochondrial damage or dysfunction caused by mitochondrial Ca^2+^ overload (Iyer et al., 2013; Miao et al., 2014; Zhuang et al., 2015), lysosomal disruption (Hornung et al., 2008; Sheedy et al., 2013; Tseng et al., 2013), autophagic dysfunction (Cho et al., 2014; Shao et al., 2014; Jabir et al., 2015) and the activity of thioredoxin-interacting protein (TXNIP; Li et al., 2015; Liu et al., 2015).

### The NLRP3 Inflammasome in Disease

While the innate immune response to insults can efficiently protect against disease and death, inappropriate activation of the NLRP3 inflammasome can contribute to the onset and progression of various diseases, particularly age-related diseases such as metabolic disorders and metabolic syndrome (Franceschi et al., 2000; Goldberg and Dixit, 2015). Increased production of IL-1β and IL-18 by the NLRP3 inflammasome contributes to atherosclerotic plaque progression and instability in atherosclerotic patients and animal models (Altaf et al., 2015; Patel et al., 2015; Peng et al., 2015). For example, Patel et al. (2015) showed that genetic ablation of the NLRP3 inflammasome suppressor known as the inhibitor of κB kinase epsilon (IKBKE) enhanced the acute phase response and down-regulated cholesterol metabolism in cultured macrophages and hypercholesterolemic mice. Atherosclerosis and other inflammatory diseases were more severe in animals with the ablation.

Studies in macrophages and animal models have shown that oxidized low-density lipoprotein and cholesterol crystals trigger NLRP3 inflammasome activation (Duewell et al., 2010; Liu et al., 2014). In macrophage and animal models of type II diabetes, hyperglycemia, and free fatty acids trigger inflammasome activation, which harms glucose metabolism and strengthens insulin resistance (Honda et al., 2014; Legrand-Poels et al., 2014; Ruscitti et al., 2015). In macrophage and animal models of uric acid accumulation, monosodium urate crystals activate the NLRP3 inflammasome, causing gout (Hari et al., 2014; Wang et al., 2014; Cleophas et al., 2015). Taken together, these findings suggest that during the progression of many metabolic diseases, the accumulation of abnormal metabolic products activates the NLRP3 inflammasome. Studies in animal models suggest a similar picture in Alzheimer’s disease (Vajjhala et al., 2012; Schnaars et al., 2013; Cho et al., 2014) and obesity induced by a high-fat diet (Haneklaus and O’Neill, 2015; Zhang et al., 2015).

In macrophages and in animal models, studies have also defined a role for the NLRP3 inflammasome in the initiation and development of cerebral and myocardial ischemic diseases, including cerebral ischemia/stroke and myocardial ischemia (Sandanger et al., 2013; Marchetti et al., 2014; Hecker et al., 2015; Ito et al., 2015). Inflammasome activation appears to contribute to post-ischemic inflammation after stroke. For example, Ito et al. (2015) showed that using ibrutinib to inhibit Bruton’s tyrosine kinase (BTK), an essential component of the NLRP3 inflammasome, reduced infarct volume, and neurological damage in a mouse model of cerebral ischemia/reperfusion injury. In addition, it is reported by Hecker et al. (2015) that activation of nicotinic acetylcholine receptors containing subunits α7, α9, and/or α10 inhibited ATP-mediated IL-1β release by human and rat monocytes, helping protect them from collateral damage. NLRP3 inflammasome-related proteins are up-regulated in myocardial fibroblasts following infarction, and this up-regulation may contribute to infarct size in ischemia-reperfusion injury (Sandanger et al., 2013). Consistent with this idea, inhibiting the NLRP3 inflammasome reduces myocardial injury after ischemia-reperfusion in mice (Marchetti et al., 2014).

NLRP3 inflammasome activation has also been linked to various auto-immune and auto-inflammatory diseases. Work from our laboratory and others has demonstrated that NLRP3 inflammasome activation contributes to progression of multiple sclerosis in humans and experimental autoimmune encephalomyelitis (EAE) in animal models (Ming et al., 2002; Jha et al., 2010; Lalor et al., 2011; Inoue et al., 2012a,b; Shao et al., 2014). Severity of multiple sclerosis in patients correlates closely with levels of IL-1β, IL-18, and caspase-1 (Ming et al., 2002; Jha et al., 2010; Lalor et al., 2011); the serum levels of both ILs and of active caspase-1 (p20) are elevated in mice with EAE (Inoue et al., 2012a,b). Studies in macrophages and mouse models of colitis have linked abnormal NLRP3 inflammasome activation with inflammatory bowel disease, including ulcerative colitis and Crohn’s disease (Cheng et al., 2015; Guo et al., 2015; Sun et al., 2015). Polymorphism in the *NLRP3* gene is linked to colitis severity and progression in patients (Villani et al., 2009; Lewis et al., 2011), and gain-of-function mutations in the *NLRP3* gene that increase production and secretion of IL-1β and IL-18 are associated with cryopyrin-associated periodic fever syndrome (CAPS; Bozkurt et al., 2015; Carta et al., 2015; Zhou et al., 2015). This syndrome comprises several rare hereditary auto-inflammatory diseases in humans and animal models, including familial cold auto-inflammatory syndrome and Muckle–Wells syndrome. Inhibiting IL-1 using specific blocking agents effectively reduces systemic inflammation in CAPS patients (Kuemmerle-Deschner, 2015; Yadlapati and Efthimiou, 2015).

## Pharmacological Use Of Nlrp3 Inflammasome Inhibitors

The extensive involvement of the NLRP3 inflammasome in such a range of diseases makes it a highly desirable drug target. Fortunately numerous promising inhibitors of NLRP3 inflammasome activation have been described, several of which are briefly described below together with their pharmacological mechanisms (shown in **Table 1**).

**Table 1:** Potential mechanisms of several NLRP3 inflammasome inhibitors.

NLRP3 inflammasome inhibitor		Potential mechanisms involving NLRP3 inflammasome inhibition
Small-molecule inhibitor	MCC950	Blocking apoptosis-associated speck-like protein (ASC) oligomerization, Inhibiting of canonical and non-canonical NLRP3 inflammasome;
	BHB	Blocking ASC oligomerization, Inhibiting K^+^/potassium eﬄux;
Type I interferon (IFN) and IFN-β		Inducting phosporylation of STAT1, transcription factor, Inducting IL-10 production;
Autophagy inducer	Resveratrol	Inducing autophagy process, Suppressing mitochondrial damage;
	Arglabin	Inducing autophagy process, Reducing cholesterol level;
	CB2R agonist	Inducing autophagy process, Inhibiting priming step of NLRP3 inflammasome activation;
MicroRNA	MicroRNA-223	Suppressing NLRP3 protein expression.


### MCC950 and β-Hydroxybutyrate

Two small-molecule inhibitors of the NLRP3 inflammasome were described in groundbreaking reports in *Nature Medicine* this year (Coll et al., 2015; Youm et al., 2015). Coll et al. (2015) discovered that MCC950, a diarylsulfonylurea-containing compound known to inhibit caspase-1-dependent processing of IL-1β (Perregaux et al., 2001), also inhibits both canonical and non-canonical activation of the NLRP3 inflammasome. MCC950 inhibits secretion of IL-1β and NLRP3-induced ASC oligomerization in mouse and human macrophages. It reduces secretion of IL-1β and IL-18, alleviating the severity of EAE and CAPS in mouse models. Coll et al. (2015) further showed that MCC950 acts specifically on the NLRP3 inflammasome: it does not inhibit the activation of NLRP1, AIM2, or NLRC4 inflammasomes. Baker et al. (2015) have shown that MCC950 inhibits LPS-induced production of IL-1β via a mechanism dependent on the cytoplasmic LPS sensors caspase-4 and caspase-5. Krishnan et al. (2015) demonstrated that hypertension in mice treated with salt and deoxycorticosterone acetate can be reversed by treating them with MCC950, and this reversal depends on the inhibition of inflammasome activation and inflammasome-related IL-1β production.

Youm et al. (2015) discovered that the ketone metabolite β-hydroxybutyrate (BHB), but not acetoacetate or the short-chain fatty acids butyrate and acetate, reduced IL-1β, and IL-18 production by the NLRP3 inflammasome in human monocytes. Like MCC950, BHB appears to block inflammasome activation by inhibiting NLRP3-induced ASC oligomerization. Their *in vivo* experiments showed that BHB or a ketogenic diet alleviate caspase-1 activation and caspase-1-mediated IL-1β production and secretion, without affecting the activation of NLRC4 or AIM2 inflammasomes. BHB inhibits NLRP3 inflammasome activation independently of AMP-activated protein kinase, ROS, autophagy, or glycolytic inhibition. These studies raise interesting questions about interactions among ketone bodies, metabolic products, and innate immunity. BHB levels increase in response to starvation, caloric restriction, high-intensity exercise, or a low-carbohydrate ketogenic diet (Cotter et al., 2013). Vital organs such as the heart and brain can exploit BHB as an alternative energy source during exercise or caloric deficiency. Future studies should examine how innate immunity, particularly the inflammasome, is influenced by ketones and other alternative metabolic fuels during periods of energy deficiency (Shido et al., 1989; Johnson et al., 2007; McGettrick and O’Neill, 2013; Mercken et al., 2013; Newman and Verdin, 2014).

Although both MCC950 and BHB inhibit NLRP3 inflammasome activation, their mechanisms differ in key respects. BHB inhibits K^+^ eﬄux from macrophages, while MCC950 does not. MCC950 inhibits both canonical and non-canonical inflammasome activation, while BHB affects only canonical activation. Nevertheless both inhibitors represent a significant advance toward developing therapies that target IL-1β and IL-18 production by the NLRP3 inflammasome in various diseases (Netea and Joosten, 2015).

### Type I Interferon (IFN) and IFN-β

In contrast to these newly described, NLRP3-specific inflammasome inhibitors, type I interferons (IFNs), including IFN-α and IFN-β, have been used for some time to inhibit the NLRP3 and other inflammasomes in various auto-immune and auto-inflammatory diseases. These diseases include multiple sclerosis, systemic-onset juvenile idiopathic arthritis caused by gain-of-function *NLRP3* mutations, rheumatic diseases and familial-type Mediterranean fever (Guarda et al., 2011a; Inoue et al., 2012b; Inoue and Shinohara, 2013b; Malhotra et al., 2015; van Kempen et al., 2015). Type I IFNs are produced by specialized immune cells such as macrophages and DCs in response to extracellular stimuli such as bacteria and virus as well as various environmental irritants (Meylan et al., 2006). These IFNs are recognized by the type I IFN receptor (IFNAR), which is a member of the TLR family and is composed of the subunits IFNAR1 and IFNAR2. IFNAR activation involves several proteins, including Janus kinases, tyrosine kinase 2, and several kinds of signal transducers and activators of transcriptions (STATs). However, how type I IFNs affect NLRP3 inflammasome and its production of IL-1β and IL-18 remains unclear (Guarda et al., 2011a), despite numerous studies aimed to improve IFN-based treatments of NLRP3 inflammasome-related diseases. To provide an example of progress in this area, we focus below on studies of IFN therapy against multiple sclerosis in patients and EAE in mice, since type I IFN therapy has been used as a first-line or standard treatment of multiple sclerosis for 15 years (Inoue et al., 2012b).

Malhotra et al. (2015) classified 97 patients with multiple sclerosis into those who responded to IFN-β therapy and those who did not, based on clinico-radiological criteria at 12 and 24 months of treatment. They found that expression of NLRP3 protein and levels of IL-1β were significantly lower among responsive patients who had relapsing-remitting multiple sclerosis than among other patients. Guarda et al. (2011a) found that IL-1β production by primary monocytes was lower in multiple sclerosis patients on IFN-β treatment than in healthy subjects, supporting the value of IFN-β therapy. Studies in mouse bone marrow-derived macrophages by Guarda et al. (2011a) suggest that IFN-β may inhibit IL-1β production through at least two mechanisms. In one pathway, phosphorylation of STAT1 transcription factor leads to repression of NLRP1 and NLRP3 inflammasomes, which in turn inhibits caspase-1-dependent IL-1β maturation. In the second pathway, type I IFNs induce IL-10 production via a STAT-dependent mechanism, and the IL-10 works in an autocrine fashion to reduce levels of pro-IL-1α and pro-IL-1β via a mechanism dependent on STAT3 signaling.

Type I IFN treatment is not effective for all types of multiple sclerosis, and the NLRP3 inflammasome may be a key determinant. Inoue et al. (2012b) conducted studies on mouse primary macrophage cultures as well as EAE mice and concluded that IFN-β therapy is effective only when the NLRP3 inflammasome contributes directly to the disease process. Their studies further showed that IFNAR activation could be inhibited using the suppressor of cytokine signal 1 (SOCS1), which inhibited Rac1 activation and ROS generation, leading in turn to inhibition of NLRP3 inflammasome activity and less severe EAE.

These studies highlight the efficacy of type I IFN therapy and the need for future studies to elucidate the mechanisms of NLRP3 inflammasome inhibition. This work may improve clinical approaches to treating multiple sclerosis and other auto-immune and auto-inflammatory diseases.

### Other Kinds of NLRP3 Inflammasome Inhibitors

Several additional ways for inhibiting the NLRP3 inflammasome have opened up in recent years. Autophagy, a self-protective catabolic pathway involving lysosomes, has been shown to inhibit the NLRP3 inflammasome, leading researchers to explore the usefulness of autophagy-inducing treatments (Shao et al., 2014). Chang et al. (2015) showed that the plant polyphenolic compound resveratrol, known to induce autophagy, suppresses mitochondrial damage in macrophages and thereby inhibits NLRP3 inflammasome activation and NLRP3 inflammasome-mediated IL-1β secretion and pyroptosis. Abderrazak et al. (2015a) showed that arglabin inhibits the production and secretion of IL-1β and IL-18 by the NLRP3 inflammasome in a concentration-dependent manner in *ApoE*^-/-^ mice on a high-fat diet. The reduced IL production translates to less severe atherosclerosis. Those authors reported that arglabin exerts its effects in macrophages by inducing autophagy as well as by reducing inflammation and cholesterol levels.

Cannabinoid receptor 2 (CB2R) is an already demonstrated therapeutic target in inflammation-related diseases (Smoum et al., 2015). Work from our own laboratory (Shao et al., 2014) has shown that autophagy induction may help explain why activation of the anti-inflammatory CB2R leads to inhibition of NLRP3 inflammasome priming and activation in mouse BV2 microglia stimulated with LPS and ATP as well as in a mouse model of EAE. Such CB2R activation reduces the severity of EAE in mice. Thus CB2R agonists similar to the HU-308 used in our work may become an effective therapy for treating NLRP3 inflammasome-related diseases by inducing autophagy.

MicroRNAs may provide another route for inhibiting inflammasomes. These endogenous non-coding RNAs are 20–23 nt long and bind to the 3′ untranslated region (3′ UTR) of protein-coding mRNAs to regulate their translation (Bartel, 2009; Chen and Sun, 2013). MicroRNA-223 binds to a conserved site in the 3′ UTR of the NLRP3 transcript, suppressing protein expression and thereby inhibiting NLRP3 inflammasome priming and IL-1β production (Bauernfeind et al., 2012; Haneklaus et al., 2012; Chen and Sun, 2013). Deficiency in microRNA-223 leads to neutrophilia, spontaneous lung inflammation, and increased susceptibility to endotoxin challenge in mice (Johnnidis et al., 2008; Haneklaus et al., 2013). Several other microRNAs have been reported to be involved in the activation of the NLRP3 inflammasome, including microRNA-155, microRNA-377, and microRNA-133a-1. Reducing the levels of these factors may be useful for treating inflammasome-related disease (Bandyopadhyay et al., 2013; Chen et al., 2015; Wang et al., 2015).

## Conclusion

The past decade has witnessed tremendous progress in understanding the structure and activation of the NLRP3 inflammasome, as well as its roles in the initiation and progression of various auto-immune and auto-inflammatory diseases, including metabolic disorders, multiple sclerosis, inflammatory bowel syndrome, and CAPS. Several types of NLRP3 inflammasome inhibitors have been developed and validated in cell culture studies and animal models of NLRP3 inflammasome-related diseases, and type I IFNs have become well established in the clinic. On the other hand, several agents have proven ineffective in clinical settings, and several potential inhibitors require further development, such as autophagy-inducing and microRNA agents. This highlights the need for further research into what pathways activate the NLRP3 inflammasome and can therefore be targeted by appropriate inhibitors. There is still a long way to go toward exploiting NLRP3 inflammasome inhibitors in our fight against diseases.

## Author Contributions

B-ZS and Z-QX were in charge of searching all the relative papers and writing this manuscript. B-ZH was in charge of drawing the picture. CL gave her valuable and professional suggestions and guide in organizing and drafting this manuscript. D-FS helped to revise the manuscript.

## Conflict of Interest Statement

The authors declare that the research was conducted in the absence of any commercial or financial relationships that could be construed as a potential conflict of interest.
